# Identification and characterization of cancer stem cells in human head and neck squamous cell carcinoma

**DOI:** 10.1186/1471-2407-14-173

**Published:** 2014-03-11

**Authors:** Jing Han, Toshio Fujisawa, Syed R Husain, Raj K Puri

**Affiliations:** 1 Tumor Vaccines and Biotechnology Branch, Division of Cellular and Gene Therapies, Center for Biologics Evaluation and Research, Food and Drug Administration, NIH Bldg 29B, Rm 2NN20, 29 Lincoln Dr., Bethesda, MD, 20892, USA; 2 Current address: Department of Gastroenterology, NTT Medical Center Tokyo, Tokyo, Japan

**Keywords:** HNSCC (head & neck squamous cell carcinoma), Stem-like cells, CD24, CD44, Salivary gland malignant neoplasms

## Abstract

**Background:**

Current evidence suggests that initiation, growth, and invasion of cancer are driven by a small population of cancer stem cells (CSC). Previous studies have identified CD44+ cells as cancer stem cells in head and neck squamous cell carcinoma (HNSCC). However, CD44 is widely expressed in most cells in HNSCC tumor samples and several cell lines tested. We previously identified a small population of CD24+/CD44+ cells in HNSCC. In this study, we examined whether this population of cells may represent CSC in HNSCC.

**Methods:**

CD24+/CD44+ cells from HNSCC cell lines were sorted by flow cytometry, and their phenotype was confirmed by qRT-PCR. Their self-renewal and differentiation properties, clonogenicity in collagen gels, and response to anticancer drugs were tested *in vitro*. The tumorigenicity potential of CD24+/CD44+ cells was tested in athymic nude mice *in vivo*.

**Results:**

Our results show that CD24+/CD44+ cells possessed stemness characteristics of self-renewal and differentiation. CD24+/CD44+ cells showed higher cell invasion in vitro and made higher number of colonies in collagen gels compared to CD24-/CD44+ HNSCC cells. In addition, the CD24+/CD44+ cells were more chemo-resistant to gemcitabine and cisplatin compared to CD24-/CD44+ cells. *In vivo*, CD24+/CD44+ cells showed a tendency to generate larger tumors in nude mice compared to CD24-/CD44+ cell population.

**Conclusion:**

Our study clearly demonstrates that a distinct small population of CD24+/CD44+ cells is present in HNSCC that shows stem cell-like properties. This distinct small population of cells should be further characterized and may provide an opportunity to target HNSCC CSC for therapy.

## Background

Squamous cell carcinoma of head and neck (HNSCC) is a heterogeneous disease [1]. Although recent advances in treatment have improved quality of life, overall 5 year survival rates have not improved significantly [2]. HNSCC frequently shows local recurrence and metastasis after the initial treatment of the primary tumor [3]. Mortality from this disease remains high because of the development of metastases and therapy-resistant local and regional recurrences [1]. Progress in treatment and prognosis for HNSCC has been limited and the molecular mechanisms of HNSCC escape from chemo- and/or radiation therapies remain mostly unknown.

Recent evidence suggests that small populations of tumor-initiating cells or cancer stem cells (CSC) are responsible for initiation, tumorigenesis, progression, and metastasis [4]. CSCs undergo self-renewal and differentiation to yield phenotypically diverse non-tumorigenic and tumorigenic cancer cells [4,5]. CSCs have been identified, isolated, and characterized in various types of cancers, such as leukemia [6], brain tumor [7], colorectal cancer [8], ovarian cancer [9], bladder cancer [10], pancreatic cancer [11] and others. It has been postulated that CSCs within the bulk tumor may escape conventional therapies, thus leading to disease relapse. Therefore, an important goal of therapy could be to identify and kill this CSC population. If CSCs can be identified prospectively and isolated, then we should be able to identify new diagnostic markers and potential therapeutic targets.

HNSCCs are heterogeneous in cellular composition. A CD44+ subpopulation of cells with CSC properties was first identified in HNSCC [12]. These CD44+ cells express a high level of the BMI1 gene, which has been demonstrated to play a role in self-renewal and tumorigenesis [13,14]. In addition to CD44, other putative stem cell markers reported to be present in HNSCC cell lines include CD29 and CD133, but the proportion of cells expressing these markers differed from one cell line to the other [15]. Additional studies indicate that ALDH activity may represent a more specific marker for CSCs in HNSCC [16,17]. It is unknown if cancer stem cell markers are tumor specific for the tissue of origin or for the niche where the tumor is growing [18].

The CD24 gene has raised considerable interest in tumor biology. A large body of literature suggests a role for CD24 in tumorigenesis and tumor progression. CD24 expression causes the acquisition of multiple cellular properties associated with tumor growth and metastasis [19]. Recent studies have identified CD24 as a marker in cancer stem cells in several cancers, including pancreatic cancer [11], colorectal cancer-derived cell lines [8], and ovarian cancer [9]. Cancer stem cell immunophenotype studies in oral squamous cell carcinoma indicated that patients with CD24 and CD44 double-positive cells showed the lowest overall survival rate compared to other immunophenotypes [20]. In our previous studies, we also found that a small population of CD24+/CD44+ cells existed in HNSCC [21]. Whether or not CD24+/CD44+ cells represent a potential phenotype of cancer stem cells in HNSCC remains to be determined.

In the present study, we have isolated the CD24+/CD44+ population from HNSCC cell lines and determined whether this cell population has cancer stem cell properties by a variety of different approaches. We demonstrate that the CD24+/CD44+ population indeed has CSC properties in HNSCC and this population should be further characterized.

## Methods

### Cell cultures

HNSCC cell line A253 (ATCC^®^HTB-41) was obtained from American Type Culture Collection (ATCC, Manassas, VA). HNSCC cell line KCCT873 was obtained from Yokohama City University Hospital [22]. A253 cells were established from tumor originated from submaxillary salivary gland. KCCT873 cells were originated from tongue tumor. A253 cells were grown in McCoy’s Modified Medium, and KCCT873 cells in RPMI 1640 medium. Cell culture media were supplemented with 10% fetal bovine serum and 1% penicillin/streptomycin (Lonza, Walkersville, MD). The cells were maintained at 37°C in a humidified atmosphere containing 5% CO_2_.

### Fluorescent-activated cell sorting and flow cytometry analysis

Cell sorting by flow cytometry was performed by Mr. Howard Mostowski at the Flow Cytometry Core facility, Center for Biologics Evaluation and Research, FDA. Cells were labeled with mouse anti-human CD44-PE (Millipore, Temecula, CA) and mouse anti-human CD24-FITC (Santa Cruz Biotech, Santa Cruz, CA) antibodies. The top or bottom cells in the 0.5 to 1 percentile fluorescence intensity of each CD24+/CD44+ and CD24-/CD44+ subpopulations were sorted and collected separately for further experiments.

For flow cytometric analysis of other markers, cells (10^6^ cells/ml) were resuspended and incubated with various antibodies, CD29-APC, CD73-APC, and CD90-PerCP-Cy5.5 (eBioscience - http://www.ebioscience.com), CD24-FITC (Santa Cruz Biotech), and CD44-PE (Millipore), according to the manufacturer’s instructions for 30 min on ice, washed with PBS three times, and fixed with 1% paraformaldehyde for later analysis. For controls, relevant isotype control antibody (eBioscience) and no antibody was used in parallel. Data were analyzed using FlowJo software (Tree Star Inc., Ashland, OR).

### Real-time PCR

For qRT-PCR, total RNAs was extracted by Trizol reagent according to the manufacturer’s instructions (Invitrogen, Carlsbad, CA). The 1^st^ strand cDNA was synthesized from 1 μg of total RNA using Superscript II Reverse Transcriptase (Invitrogen) according to manufactures specifications. The resulting cDNA was amplified by using gene-specific primers. The primer sequences for each tested gene are listed in Additional file 1: Table S1. For amplification, samples were prepared with SsoAdvanced^TM^ SYBR^®^ Green Supermix (Bio-Rad) following the manufacture’s protocol, and run on a Bio-Rad CFX96 Touch^TM^ Real-Time Detection System. Buffer only and no template were included in each assay run as controls. All samples and controls were run in triplicate. Gene-specific amplification was normalized to β-Actin and relative fold change was calculated following the manufacture’s protocol (Bio-Rad).

### Cell proliferation assay

One thousand sorted cells per well were cultured in quadruplicate in 96-well plates for the indicated period of time. Cell proliferation was detected by using CellTiter-Glo® Luminescent Cell Viability Assay kit (Promega, Madison, WI). Cell viability was quantified by measuring the absorbance using a microplate reader (Molecular Devices, Sunnyvale, CA) with 500 ms integration. Experimental background was determined by using empty wells with medium.

### Colony-forming assay

Collagen type I gels were prepared with cell culture medium to make final collagen concentration of 2 mg/mL (pH = 7.0) [23]. For cell cultures within collagen gels, 1.5 mL cell suspension (500 cells/mL) was mixed with 1.5 mL of collagen solution. The mixture was plated in six-well plates, and placed in 37°C incubator for gelation. After gelation, the collagen gels were overlaid with 3 mL of complete medium and incubated in a humidified atmosphere containing 95% air and 5% CO_2_. Cells were cultured for six days. Cell colonies were visualized with Coomassie Blue solution staining (0.5% Coomassie Brilliant Blue G250, Bio-Rad), and visible colonies were counted. Assays were performed in triplicate.

### Matrigel invasion assay

Cell invasion was studied by using BD BioCoat Matrigel invasion chambers (BD Biosciences; 24-well, 8 μm pore size) with 10% fetal bovine serum as a chemo attractant, and following the manufacture’s protocol. Briefly, one thousand cells were loaded into the chamber and incubated for 24 to 72 hrs at 37°C. Noninvasive cells were removed from the upper surface of the membrane with a cotton swab, and cells on the bottom surface of the membrane were fixed and stained with H&E. Cells in five random fields per well were counted. The experiments were performed in duplicate.

### Drug sensitivity assay

Following cell sorting, both CD24+/CD44+ and CD24-/CD44+ cells were cultured for 2 days to eliminate damaged cells caused by the sorting process. Cells were then plated at a density of 1 × 10^3^/well in 96-well plates. Chemotherapeutic reagents, Gemcitabine or Cisplatin, were added to the cells at gradually increasing concentrations. The cells were cultured for 72 hrs, and the cell viability was determined by CellTiter-Glo^®^ Assay (Promega, Madison, WI) according to the manufacturer’s protocol.

### Tumor xenograft studies

Animal studies were conducted under a CBER ACUC-approved protocol in accordance with the principles and procedures outlined in *the NIH Guide for the Care and Use of Laboratory Animals*. Female athymic nude immunodeficient mice between 4-to 6-week-age were obtained from the NCI Animal Facility (NCI-Frederick). Before injection, cells were re-suspended in a 1:1 mixture of Matrigel (BD Biosciences) and PBS. A 100-μl cell suspension containing 100, 1,000, or 10,000 sorted CD24+ and CD24-cells was subcutaneously injected into the dorsal flank of each mouse. For the control groups, mice received 100 μl injections of the parent unsorted cells in corresponding concentrations. Tumor size (major axis × the minor axis) was measured weekly after tumor challenge. Animal experiments were repeated several times. At the end of the experimental period, tumor tissues were collected and fixed in formalin for further immunohistochemical studies.

### Immunohistochemical studies of HNSCC tumor tissues

Immunohistochemical (IHC) studies of tumor sections were performed on formalin-fixed, paraffin-embedded tumors isolated from tumor xenografts in the study. Tissue sections were deparaffinized by xylene, and re-hydrated with sequential washes of 100%, 75%, and 50% ethanol, and PBS. For antigen retrieval, slides were placed in 50 mM citrate buffer pH 6.0 (Vector Lab, CA), boiled for 5 min, and stayed in the buffer for 15 min. Endogenous peroxidase activity was inhibited with 3% hydrogen peroxidase in PBS. Non-specific binding was blocked with 2.5% normal serum and 1% bovine serum albumin (BSA) for 1 hr. Tissue sections were incubated with various antibodies, CD24 and CD44 (Millipore), or isotype control (IgG) (Sigma) overnight at 4°C. Immunodetection was performed using ABC staining systems according to manufacturer’s instructions (Santa Cruz Biotech). All sections were counterstained with haematoxylin. After dehydration with washes of 95% and 100% ethanol and xylene, tissue sections with permanent mounting medium were covered with glass coverslips, and viewed by light microscope. H&E staining was also performed on the section from each tumor tissue sample.

### Statistical analysis

Statistical analyses were performed by paired Student’s t-test between two groups. Data were presented as mean ± SD. P value of < 0.05 was considered statistically significant. Each experiment was repeated at least twice including animal experiments.

## Results

### Isolation and characterization of CD24+/CD44+ cells in HNSCC cell lines

**Figure 1:**
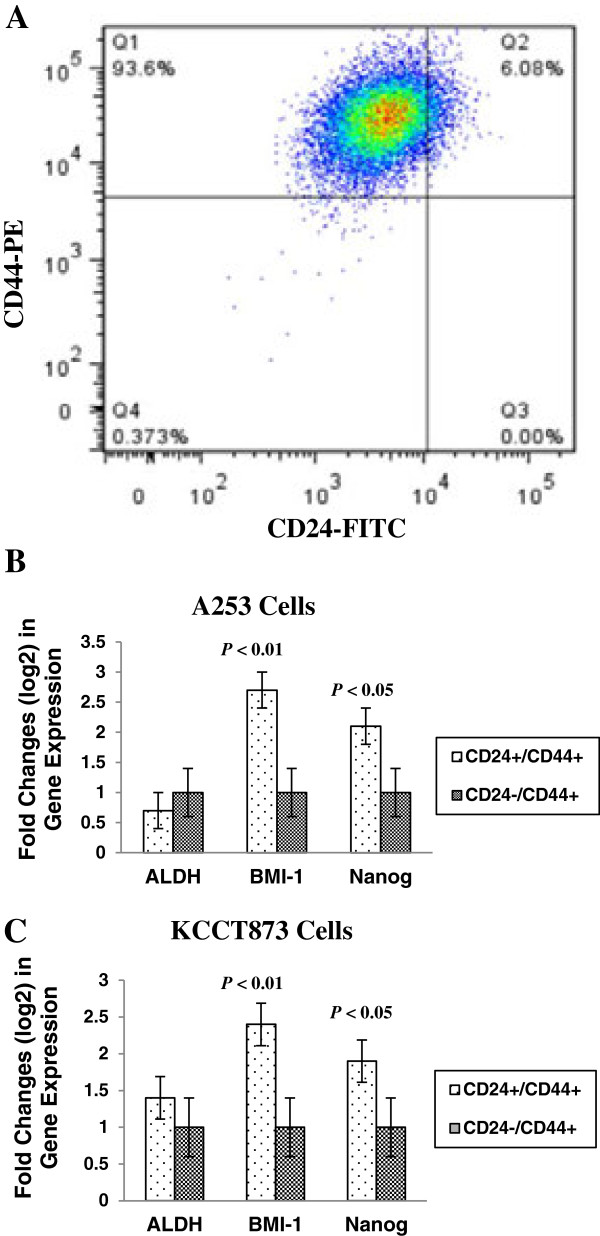
**Expression of CD24 and CD44 in A253 HNSCC cells. (A)** Flow cytometric analysis of CD24+ and CD44+ cells in A253 HNSCC cell line. Dual staining of A253 HNSCC cells indicate that CD24+/CD44+ subpopulation is ~6%, while CD24-/CD44+ subpopulation is >93% in the whole cell population. qRT-PCR analysis of stemness-related genes in FACS-sorted CD24+/CD44+ and CD24-/CD44+ cells derived from A253 **(B)** and KCCT873 **(C)** tumor cells. Data represent log2 mean fold changes in gene expression ± SD of triplicate determinations in CD24+/CD44+ compared to CD24-/CD44+ subpopulations from both cell lines. *P* values for two genes, BMI1 and Nanog, in two cell lines are shown.

To determine the percentage of the putative cancer stem-like cells in the HNSCC cell population, cell suspensions from cell lines A253 and KCCT873 were analyzed and sorted for cell surface markers CD24 and CD44 by flow cytometry. Two phenotypic subpopulations were separated. CD24+/CD44+ cells were only ~5-8% in whole cell population. In contrast, CD24-/CD44+ cells were >90% in whole cell population of both HNSCC cell lines (Figure 1A).

We next investigated the expression of known “stemness” genes in the isolated CD24+/CD44+ and CD24-/CD44+ subpopulations by real-time RT-PCR technology. We tested expression of six genes including ALDH1, BMI1, CD133, Nanog, Oct3/4, and Sox2. BMI1 and Nanog genes showed a significantly higher expression in CD24+/CD44+ compared to CD24-/CD44+ subpopulations from both HNSCC cell lines. However, there was no significant difference in ALDH1 expression between CD24+/CD44+ and CD24-/CD44+ subpopulations from both cell lines (Figure 1B and C). CD133 was only expressed in one cell line (KCCT873) at a very low level and did not show a clear difference between two subpopulations of cells (data not shown). A253 cells did not show any expression of CD133 gene. The expression of Oct3/4 and Sox2 was absent in both cell subpopulations in both cell lines (data not shown).

### Cellular properties of CD24+/CD44+ cells *in vitro*

**Figure 2:**
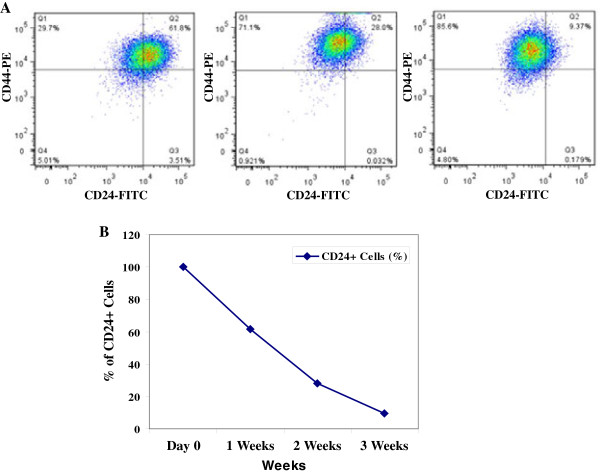
**Differentiation of CD24+/CD44+ cells. (A)** A253 CD24+ HNSCC cells differentiate into CD24-cells. Population dynamics modeled by a simple growth model in which CD24+ cells divide and switch to a CD24-state. Flow cytometry plots illustrate the sorted CD24+ cell populations at week one, two and three, from left to right panels. **(B)** Flow sorted CD24+ cells were monitored for 3 weeks in cell culture for their ability to convert into CD24-cells. Day 0 indicates the day cells were sorted by CD24 expression. The percentage of the CD24+ cells decreased in a time-dependent manner.

To explore the self-renewal and differentiation capacity of CD24+/CD44+ cells, the purified CD24+/CD44+ cells were cultured *in vitro* for 3 weeks, and variations in CD24 expression were examined by flow cytometry. We found that the proportion of CD24+/CD44+ cells dramatically declined in a time dependent manner in the CD24+/CD44+ sorted population of cells. CD24+ cells in CD24+/CD44+ population decreased to ~62% one week after culture and continued to decrease to 28% two weeks after cell culture. The proportion of the CD24+/CD44+ cells returned to similar presorting level (< 10%) after three weeks culture. In contrast, the proportion of CD24-/CD44+ cells in the cell population gradually increased from ~30% at the first week to ~86% after three weeks, indicating that the CD24+/CD44+ cells give rise to CD24-/CD44+ cells (Figure 2A and B).

**Figure 3:**
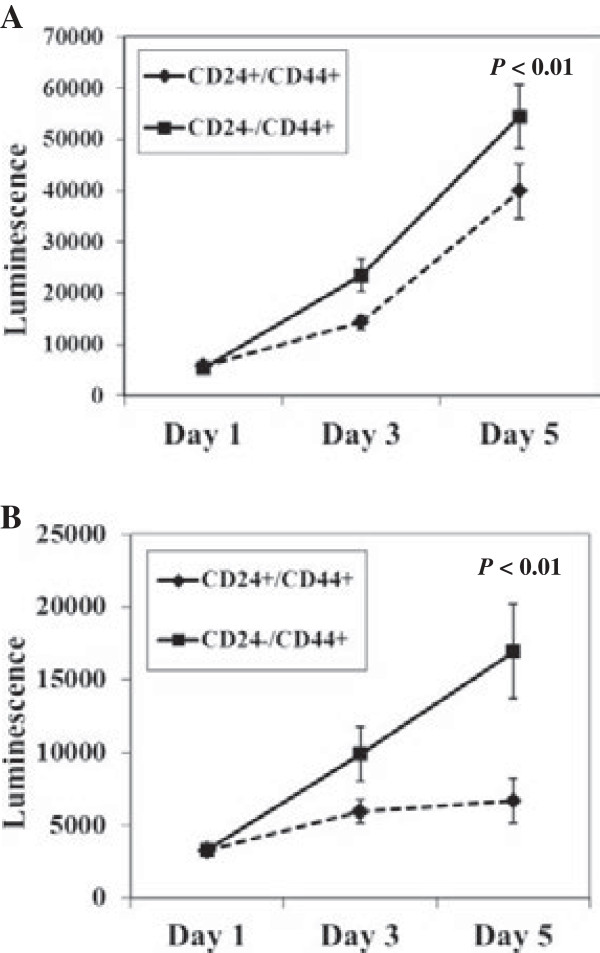
**Cell proliferation assay.** Cells were cultured in quadruplicate in a 96-well plate at a density of 1000 cells/per well, and proliferation was measured by Cell Titter-Glo^®^ cell viability assay. Growth curve of CD24+/CD44+ and CD24-/CD44+ subpopulations of A253 cells **(A)** and KCCT873 cells **(B)** are shown. Data represent mean ± SD of triplicate determinations. *P* value is shown for day 5 time point.

Cell proliferation assays indicated that the growth rate of CD24+/CD44+ cells was slightly lower compared to CD24-/CD44+ cells for up to 5 days after cell sorting (Figure 3A and B). These results indicate that CD24+/CD44+ cells show asymmetric division-like proliferation pattern, indicating the self-renewal and differentiation potential to produce heterologous descendent CD24-/CD44+ cells in culture.

**Figure 4:**
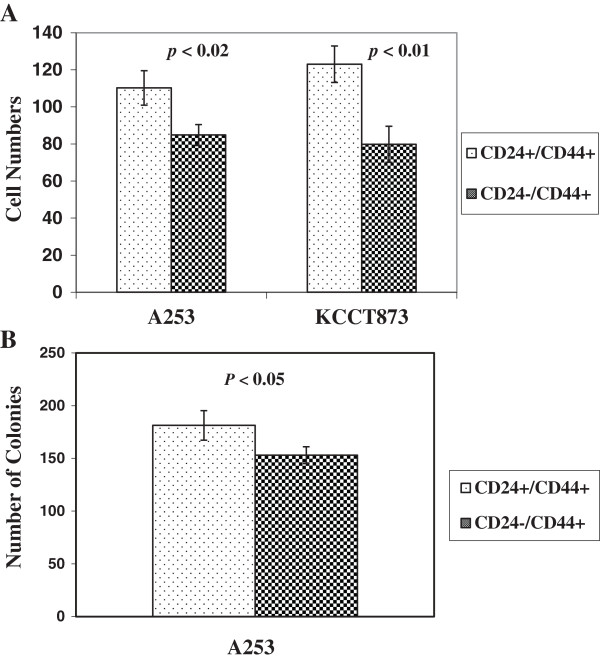
**Cell invasion and clonogenic assays. (A)** Matrigel invasion activity of CD24+/CD44+ and CD24-/CD44+ flow cytometry-sorted cells from HNSCC cell lines. The number of cells invading through the Matrigel was assessed at 24 hr. **(B)** Colony-forming assay with FACS-sorted CD24+/CD44+ and CD24-/CD44+ cells. The CD24+/CD44+ cells show significantly higher number of colonies. *P* values for invasion and clonogenic assays are shown in the figure.

We next investigated the invasion ability of CD24+/CD44+ and CD24-/CD44+ subpopulations by matrigel invasion assays. We observed that the number of invading cells in the CD24+/CD44+ cells was significantly higher compared to CD24-/CD44+ cells, indicating that CD24+/CD44+ cells have higher invasion ability compared to CD24-/CD44+ cells (p < 0.02 for A253 and p < 0.01 for KCCT873 compared to CD24-/CD44+ cells) (Figure 4A).

The colony-formation capacity of CD24+/CD44+ and CD24-/CD44+ subpopulations was also tested. Our results indicate that CD24+/CD44+ cells form significantly higher number of colonies compared to CD24-/CD44+ cell subpopulation (*p* < 0.05) (Figure 4B).

### CD24+ cells show higher drug resistance to chemotherapeutic agents *in vitro*

**Figure 5:**
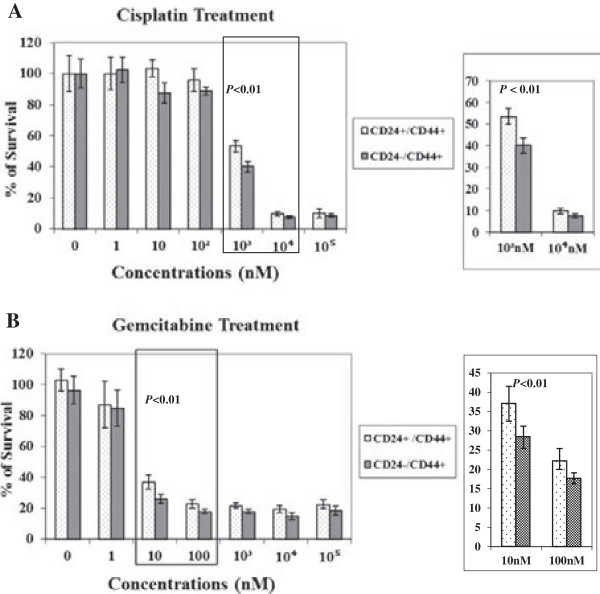
**Sensitivity of CD24+/CD44+ and CD24-/CD44+ cells to cisplatin and gemcitabine anticancer drugs.** Flow cytometry sorted cells were exposed to cisplatin **(A)**, and gemcitabine **(B)** at increasing concentrations for 72 hr, followed by cell viability measurement by Cell Titter-Glo^®^ Cell Viability Assay. Differences in drug resistance between CD24+/CD44+ and CD24-/CD44+ cells were calculated. All experiments were performed in triplicate and data are shown as mean ± SD. Data in inset show statistical significance at *p* < 0.01 for both treatments.

Cisplatin (cis-diammine-dichloroplatinum (II)) is used for treatment of a wide range of cancers, including head & neck tumors. Cisplatin often leads to an initial therapeutic success associated with partial response or disease stabilization [24]. Gemcitabine is a nucleoside analog displaying a wide spectrum of antitumor activity [25]. Although both drugs have been used for chemotherapeutic treatment of patients with head & neck tumors, many patients are intrinsically resistant to these drugs [24]. Recent studies have indicated that cancer stem cell phenotypes are associated with drug resistance to chemotherapeutic drugs [26,27]. To evaluate the drug resistance properties of FACS sorted HNSCC cells, CD24+/CD44+ and CD24-/CD44+ cells were grown and treated with various concentrations of either cisplatin or gemcitabine for 72 hours, and then cell survival was assessed by determining cell viability. CD24+/CD44+ cells seem to show small but significantly higher drug resistance to either chemotherapeutic agent when compared to CD24-/CD44+ cells (Figure 5). For example, CD24+/CD44+ cells showed higher survival rate (53.5%) compared to CD24-/CD44+ cells (40%) when treated with 1000 nM cisplatin (*p* < 0.01) (Figure 5A). Similarly, CD24+/CD44+ cells showed > 10% higher survival rate (37%) compared to survival rate (26%) of CD24-/CD44+ cells when treated with 10 nM gemcitabine (*p* < 0.01) (Figure 5B).

### Tumorigenicity of CD24+/CD44+ and CD24-/CD44+ subpopulations

**Figure 6:**
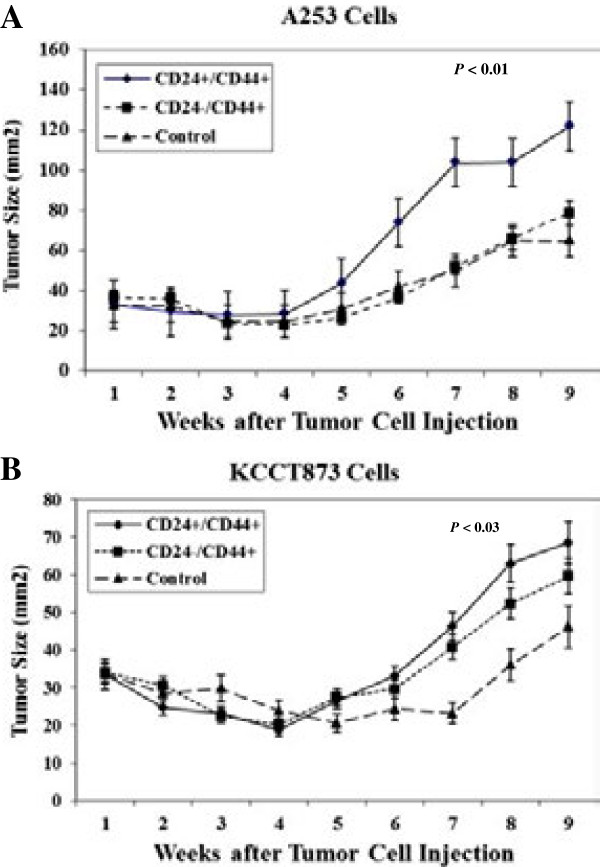
***In vivo*tumorigenicity of CD24+/CD44+ and CD24-/CD44+ HNSCC cells.** Athymic nude mice were injected s.c. 1000 cells in 100 μl matrigel containing either CD24+/CD44+, CD24-/CD44+, or unsorted cells (control). Each group had five animals and experiment was repeated several times. **(A)** Tumors were generated from A253 cells; and **(B)** KCCT873 cells. Tumor sizes were measured once a week and shown as mean ± SD. *P* value is shown for week 9 groups comparing CD24+/CD44+ and CD24-/CD44+ HNSCC tumors.

We next evaluated whether the two subpopulations (CD24+/CD44+ and CD24-/CD44+) of HNSCC cells were endowed with differential tumorigenic potential. Several independent experiments were performed with two different HNSCC cell lines. The two phenotypic subpopulations of cells, CD24+/CD44+ and CD24-/CD44+, were sorted by flow cytometry, suspended in a Matrigel mixture (1:1), and then S.C. injected into athymic nude mice. The tumor size was measured weekly for 9 weeks, at which time animals were sacrificed. When minimal (1 × 10^2^) to maximal (1 × 10^4^) numbers of cells per mouse were injected, both CD24+/CD44+ and CD24-/CD44+ cells formed tumors and thus were tumorigenic. However, the size of tumor generated by CD24+/CD44+ cells was significantly larger than the size of the tumors from CD24-/CD44+ or unsorted control cells (Figure 6) indicating CD24+/CD44+ cells are highly aggressive.

**Figure 7:**
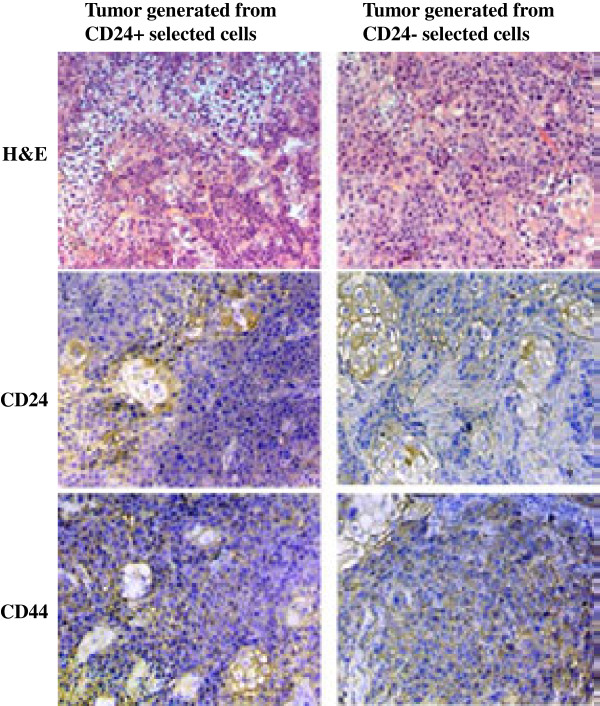
**Immunohistochemical analyses of CD24 and CD44 in tumors generated from CD24+/CD44+ and CD24-/CD44+ HNSCC cells.** Both CD24 and CD44 show cell surface staining. CD24 was only present on the salivary gland type cells and show membrane and cytoplasmic staining. CD44 show strong positive reactions not only in salivary gland type cells, but also in most tumor cells. Tumors generated from CD24+ and CD24- cells showed the similar immunohistochemical staining patterns for CD24 and CD44.

Immunohistochemical staining for CD24 and CD44 on tumor tissues isolated from tumor xenografts at the end of the study were performed to determine whether CD24+/CD44+ CSC maintained their phenotype at the end of the experiment. Upon H&E staining, A253 cells showed submaxillary salivary gland features since these cells originated from submaxillary salivary gland tumor. KCCT873 cells showed similar features. By IHC, strong positive staining for CD24 was observed on the surface of salivary gland appearing structure in xenograft tumors derived from both cell lines. In addition, strong positive staining for CD44 was observed not only on the surface of salivary gland appearing structures, but also on the dense carcinoma cells within the tumor mass as well (Figure 7). Since xenograft tumors generated from both CD24+/CD44+ and CD24-/CD44+ cells showed the similar immunohistochemical staining, we hypothesized that the CD24+/CD44+ cells may have been generated during the in vivo tumor growth from CD24-/CD44+ cell subpopulation.

### Flow cytometry analysis of additional stemness cell markers

**Figure 8:**
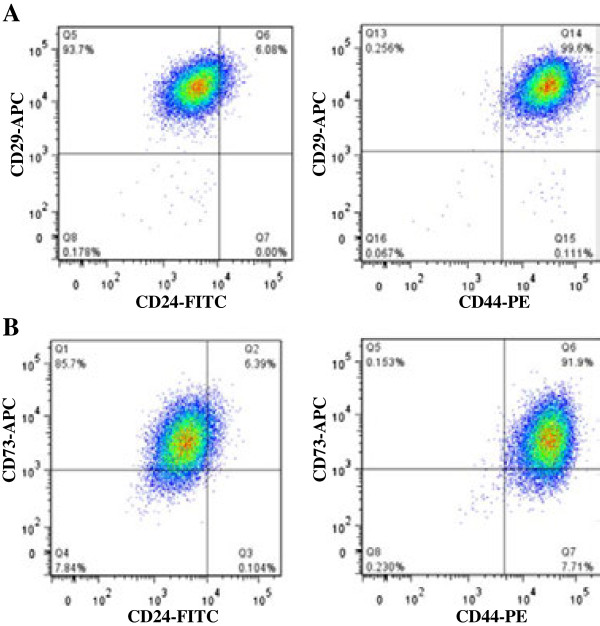
**Representative flow cytometry plots analyzing expression of CD29, CD24, CD44, and CD73 in A253 HNSCC cells. (A)** Co-expression of CD29 with CD44 and CD24. **(B)** Co-expression of CD73 with CD44 and CD24.

To investigate whether other putative stem cell markers were expressed in HNSCC cells, the mesenchymal stem cell markers, CD29 (β1-integrin), CD73 (5′-nucleotidase), CD90 (Thy-1), and CD105 (Endogin) were selected and analyzed by flow cytometry. CD29 expression showed the strongest correlation with the CD44 expression. Almost all cells (99.6%) were CD29 and CD44 double-positive. Only ~6% of the cells were CD29+ and CD24+, the same percentage found for CD24+/CD44+ cells (Figure 8A). CD73 also showed a strong correlation with CD44 expression. Approximately 92% of the cells were CD73 and CD44 double-positive, while only ~6% of the cells were CD73+/CD24+, similar to CD24+/CD44+ cells (Figure 8B). On the other hand, neither CD90 nor CD105 showed any correlation with either CD24 or CD44 expression (data not shown).

## Discussion

We have identified and characterized a distinct CD24+ subpopulation in the CD44+ population of HNSCC tumors. These CD24+/CD44+ cells derived from HNSCC cell lines displayed several features typically seen in cancer stem cells, including the ability to differentiate and self-renewal. CD24+/CD44+ cells were more proliferative and invasive *in vitro* and more tumorigenic *in vivo* forming larger tumors in immunodeficient mice compared to its counterpart CD24-/CD44+ cells. In addition, CD24+/CD44+ cells were slightly more resistant to chemotherapeutic agents compared to CD24-/CD44+ cells. These findings indicate that a distinct CD24+/CD44+ subpopulation may represent CSC or tumor initiating cells in HNSCC.

We confirmed the stemness feature of CD24+/CD44+ cells by showing that CD24+/CD44+ cells express higher levels of BMI1 and Nanog genes compared to CD24-/CD44+ cells. BMI1 has been shown to play a role in the self-renewal of hematopoietic stem cells [14] and is considered a stem cell related gene. BMI1 has also been implicated in tumorigenesis, primarily in leukemias [13], and in several human cancers including HNSCC [12]. Similarly, Nanog gene has been shown to be associated with stemness of human embryonic stem cells [28]. These results support our finding that CD24+/CD44+ cell subpopulations are indeed CSC in HNSCC. Our data also show a strong correlation between CD29 (β1-integrin) and CD44 expression in HNSCC. More than 99% cells were CD29 and CD44 double-positive, indicating CD24+/CD44+ cells were also CD29+. Recently, a subpopulation of cells (Lin^−^/CD29+/CD24+) isolated from mouse mammary cells was identified as mammary stem cells [29]. It is also reported that CD24 expression positively associated with salivary gland cancer stage III/IV [30]. These authors showed that double positive (CD24+/CD44+) cells may represent tumors with most aggressive behavior and worst prognosis [30].

Although ALDH1, CD133, Oct3/4, and Sox2 have been identified as a putative marker for cancer stem cells in many cancers including HNSCC, we did not find a significant difference of these genes between CD24+/CD44+ and CD24-/CD44+ cell populations. In addition, Oct3/4, Sox2 and CD133 were not consistently expressed in these cells. It is possible that different tumor cell lines, types and origin of tumors may have different phenotype of HNSCC CSCs.

Previous studies have demonstrated that CD24 is involved in cell adhesion and metastatic tumor spread [19,31,32], and may be one of the cancer stem cell markers expressed in various cancer cell lines [33]. Consistent with our observations, a highly tumorigenic subpopulation of cells with CD44+/CD24+/ESA + phenotype was identified as cancer stem cells in pancreatic cancer [11]. Although this phenotype was only 0.2 to 0.8% in the whole pancreatic cancer cell population, it had a 100-fold increased tumorigenic potential compared with other phenotypes [11]. Similarly, a CD24+/CD44+ cancer stem cell subpopulation has been identified in solid tumors and cancer cell lines in both colorectal and ovarian cancers [8,9]. CD24 has been shown to be related to invasiveness and differentiation of colorectal adenocarcinoma [34]. CD24 has also been identified as one of the cancer stem cell markers in human malignant mesothelioma cells [35]. These studies suggest that CD24 is both a marker of tumor aggressiveness and a promoter of metastatic tumor growth. Thus, targeting CD24 may offer new approach for therapy of human cancer including HNSCC.

Similar to CD24, previous studies have identified CD44, BMI1 and ALDH1 as putative markers for CSC in head and neck squamous cell carcinomas [12,16,17]. CD44 has also been identified as one of the CSC markers in various other cancer types [8,11,12,20,33]. CD44 was not only found to be constitutively expressed in the HNSCC cell lines, but also abundantly expressed in head and neck carcinomas [21,36,37]. HNSCC tumors can arise from many location of the upper aerodigestive tract, including the nasal cavity, sinus cavities, oral cavity, pharynx, or larynx. The various locations associated with malignant transformation implicated a wide-range of tumors representative of the anatomic locations [38]. Although multiple cell surface markers have been identified as cancer stem cell markers, it is clear that no marker can be used universally to identify cancer stem cells in HNSCC. Expression of various CSC markers shows great variations between different tumor types, even in the same tumor but different subtypes [33]. CD24+/CD44+ subpopulation identified in our study may represent a new subtype of the cancer stem cells in HNSCC, specifically in salivary gland malignant neoplasms.

It was noted that the tumors generated by both CD24+/CD44+ and CD24-/CD44+ cells were positive for CD24+/CD44+ in IHC studies. IHC staining of xenograft tumor tissues showed positive staining for CD24 on the salivary gland appearing structures. In addition, strong positive staining for CD44 was observed not only on the surface of salivary gland appearing structure, but also on the carcinoma cells within the tumor mass. There are two possible explanations for the presence of CD24+/CD44+ tumor cells from CD24-/CD44+ tumors. First, CD24+/CD44+ cells may have been generated during the *in vivo* tumor growth from CD24-/CD44+ cell population. This hypothesis is supported by recent publications that indicate that normal and neoplastic nonstem cells can spontaneously convert to a stem-like state. Chaffer et al., showed that CD44^hi^ cells can differentiate into CD44^lo^/CD24+/ESA^−^ and CD44^lo^/CD24+/ESA + progeny, and CD44^lo^ cells can spontaneously convert to CD44^hi^ cells [39]. Second, since CD24+/CD44+ and CD24-/CD44+ HNSCC cells were sorted by FACS technology, we cannot rule out the possibility of undetectable residual CD24+/CD44+ cells contaminating the CD24-/CD44+ cell population, which resulted in CD24+/CD44+ cells within the xenograft tumors, although this was considered a remote possibility.

## Conclusion

We have demonstrated that HNSCC contain a distinct CD24+/CD44+ cell subpopulation that possesses cancer stem cell-like properties. CD24+/CD44+ cells are able to self-renew, differentiate into different phenotypes, initiate and develop tumors in athymic nude mice faster. Identification of cancer stem cells may provide novel insights into the development of new therapeutic approaches for HNSCC.

## Abbreviations

HNSCC: Head and neck squamous cell carcinoma; CSC: Cancer stem cell; ALDH: Aldehyde dehydrogenase; BMI1: BMI1 polycomb ring finger oncogene; ESA: Epithelial-specific antigen.

## Competing interests

All authors declare that they have no competing interest.

## Authors’ contributions

JH designed the study, carried out the experimental work, performed data analysis and interpreted results, and drafted the manuscript. TF and SRH carried out some experimental work, collected and analyzed data, interpreted results, and edited manuscript. RKP conceived and designed the study, supervised data analysis, interpreted results, edited and revised the manuscript, and negotiated for its publication. All authors approved the submission of this version of manuscript, and assert that the document represents valid work. All contributing authors have no disclosures to make.

## Pre-publication history

The pre-publication history for this paper can be accessed here:

http://www.biomedcentral.com/1471-2407/14/173/prepub

## Supplementary Material

Additional file 1: Table S1 Selected gene primers for qRT-PCR. Click here for file

## References

[B1] Mannelli G Gallo O Cancer stem cells hypothesis and stem cells ion head and neck cancers Cancer Treat Rev 2012 38 515 539 10.1016/j.ctrv.2011.11.007 22197808

[B2] Goon PKC Stanley MA Ebmayer J Steinstrsässer L Upile T Jerjes W Bernall-Sprekelsen M Görner M Sudhoff HH HPV & head and neck cancer: a descriptive update Head Neck Oncol 2009 1 36 10.1186/1758-3284-1-36 19828033 PMC2770444

[B3] Marur S Forastiere AA Head and neck cancer: changing epidemiology, diagnosis, and treatment Mayo Clin Proc 2008 83 489 501 10.4065/83.4.489 18380996

[B4] Nguyen LV Vanner R Dirks P Eaves CJ Cancer stem cells: an evolving concept Nat Rev Cancer 2012 12 133 143 22237392 10.1038/nrc3184

[B5] Reya T Morrison SJ Clarke MF Weissman IL Stem cells, cancer, and cancer stem cells Nature 2011 414 105 111 11689955 10.1038/35102167

[B6] Huntly BJP Gilliland DG Leukaemia stem cells and the evolution of cancer-stem-cell research Nat Rev 2005 5 311 321 10.1038/nrc1592 15803157

[B7] Singh S Hawkins C Clarke ID Squire JA Bayanl J Hide T Henkelman RM Cusimano MD Dirks PB Identification of human brain tumor initiating cells Nature 2004 432 396 401 10.1038/nature03128 15549107

[B8] Yeung TM Gradhi SC Wilding JL Muschel R Bodmer WF Cancer stem cells from colorectal cancer-derived cell lines PNAS 2009 107 3722 3727 20133591 10.1073/pnas.0915135107 PMC2840416

[B9] Gao MQ Choi YP Kang S Houn JH Cho NH CD24+ cells from hierarchically organized ovarian cancer are enriched in cancer stem cells Oncogene 2010 29 2697 80 10.1038/onc.2010.35 20190812

[B10] Overdevest JB Thomas S Kristiansen G Hansel DE Smith SC Theodorescu D CD24 offers a therapeutic target for control of bladder cancer metastasis based on a requirement for lung colonization Cancer Res 2011 71 3802 3811 10.1158/0008-5472.CAN-11-0519 21482678 PMC4283788

[B11] Li C Heidt DG Dalerba P Burant CF Zhang L Adsay V Wicha M Clarke MF Simeone DM Identification of pancreatic cancer stem cells Cancer Res 2007 67 1030 1037 10.1158/0008-5472.CAN-06-2030 17283135

[B12] Prince ME Sivanandan R Kaczorowski A Wolf GT Kaplan MJ Dalerba P Weissman IL Clarke MF Ailles LE Identification of a subpopulation of cells with cancer stem cell properties in head and neck squamous cell carcinoma PNAS 2007 104 973 978 10.1073/pnas.0610117104 17210912 PMC1783424

[B13] Lessard J Sauvageaun G *Bmi-1* determines the proliferative capacity of normal and leukaemic stem cells Nature 2003 423 255 260 10.1038/nature01572 12714970

[B14] Park IK Morrison SJ Clarken MF *Bmi1*, stem cells, and senescence regulation J Clin Invest 2004 113 175 179 10.1172/JCI200420800 14722607 PMC311443

[B15] Harper LJ Piper K Common J Fortune F Mackenzie IC Stem cell patterns in cell lines derived from head and neck squamous cell carcinoma J Oral Pathol Med 2007 36 594 603 10.1111/j.1600-0714.2007.00617.x 17944752

[B16] Chen YC Chen YW Hsu HS Tseng LM Huang PI Lu KH Chen DT Tai LK Yung MC Chang SC Ku HH Chiou SH Lo WL Aldehyde dehydrogenase 1 is a putative marker for cancer stem cells in head and neck squamous cancer Biochem Biophy Res Commun 2009 385 307 313 10.1016/j.bbrc.2009.05.048 19450560

[B17] Clay MR Tabor M Owen JH Carey TE Bradford CR Wolf GT Wicha MS Prince ME Single-marker identification of head and neck squamous cell carcinoma cancer stem cells with aldehyde dehydrogenase Head Neck 2010 32 1195 1201 10.1002/hed.21315 20073073 PMC2991066

[B18] Alberts AE Chen C Koberle B Qian X Klussmann JP Wollenberg B Kaufmann AM Stem cells in squamous head and neck cancer Crit Rev Oncol/Hemoatol 2012 81 224 240 10.1016/j.critrevonc.2011.03.004 21511490

[B19] Baumann P Cremers N Kroese F Orend G Chiquet-Ehrismann R Uede T Yagita H Sleeman JP CD24 expression causes the acquisition of multiple cellular properties associated with tumor growth and metastasis Cancer Res 2005 65 10783 10793 10.1158/0008-5472.CAN-05-0619 16322224

[B20] Oliveira LR Oliveira-Costa JP Araujo IM Soave DF Zanetti JS Soares FA Zucoloto S Ribeiro-Silva A Cancer stem cell immunophentypes in oral squamous cell carcinoma J Oral Path Med 2011 40 135 142 10.1111/j.1600-0714.2010.00967.x 21073537

[B21] Han J Kioi M Chu WS Kasperbauer JL Strome SE Puri RK Identification of potential therapeutic targets in human head & neck squamous cell carcinoma Head Neck Oncol 2009 1 27 10.1186/1758-3284-1-27 19602232 PMC2719634

[B22] Kawakami K Leland P Puri RK Structure, function, and targeting of interleukin 4 receptors on human head and neck cancer cells Cancer Res 2000 60 2981 2987 10850446

[B23] Han J Daniel JC Biosynthesis of type VI collagen by glioblastoma cells and possible function in cell invasion of three-dimension matrices Connect Tissue Res 1995 31 161 170 10.3109/03008209509028404 15612332

[B24] Galluzzi L Senovilla L Vitale I Michels J Martins I Kepp O Castedo M Kroemer G Molecular mechanisms of cisplatin resistance Oncogen 2012 31 1869 1883 10.1038/onc.2011.384 21892204

[B25] Mini E Nobili S Caciagli B Landini I Mazzei T Cellular pharmacology of gemcitabine Ann Oncol 2006 17 v7 v12 10.1093/annonc/mdj941 16807468

[B26] Dean M Fojo T Bates S Tumor stem cells and drug resistance Nat Rev 2005 5 275 284 10.1038/nrc1590 15803154

[B27] Hu G Li F Ouyang K Xie F Tang X Wang K Han S Jiang Z Zhu M Wen D Qin X Zhang L Intrinsic gemcitabine resistance in a novel pancreatic cancer cell line is associated with cancer stem cell-like phenotype Int J Oncol 2012 40 798 806 22076649 10.3892/ijo.2011.1254

[B28] Bhattacharya B Milura T Brandenberger R Mejido J Luo Y Yang AX Joshi BH Ginis I Thies RS Amit M Lyons I Condie BG Itskovitz-Eldor J Rao MS Puri RK Gene expressions in human embryonic stem cell lines: unique molecular signature Blood 2004 103 2956 2964 10.1182/blood-2003-09-3314 15070671

[B29] Shackleton M Vaillsny F Simpson KJ Stingl J Smyth GK Asselin-Labbat ML Wu L Lindeman GJ Visvader JE Generation of a functional mammary gland from a single stem cell Nature 2006 439 84 88 10.1038/nature04372 16397499

[B30] Soave DF da Costa JPO de Silveira GG Ianez RC de Oliveira LR Lourenco SV Ribeiro-Silva A CD44/CD24 immunophenotypes on clinicopathologic features of salivary glands malignant neoplasms Diagn Pathol 2013 8 29 10.1186/1746-1596-8-29 23419168 PMC3605183

[B31] Kristiansen G Schluns K Yongwei Y Denkert C Dietel M Peterson I CD24 expression is a new prognostic marker in breast cancer Clin Cancer Res 2003 9 4906 4913 14581365

[B32] Kristiansen G Pilarsky C Pervan J Stürzebecher B Stephan C Jung K Loening S Rosenthal A Dietel M CD24 expression is a significant predictor of PSA relapse and poor prognosis in low grade or organ confined prostate cancer Prostate 2004 58 183 192 10.1002/pros.10324 14716744

[B33] Stuelten CH Mertins S Busch JI Gowens M Scudiero DA Burkett MW Hite KM Alley M Hollingshead M Shoemaker RH Niederhuber JE Complex display of putative tumor stem cell markers in the NCI60 tumor cell line panel Stem Cell 2010 28 649 660 10.1002/stem.324 PMC7444750 20178109

[B34] Choi D Lee HW Hur KY Kim JJ Park GS Jang SH Song YS Jang KS Paik SS Cancer stem cell markers CD133 and CD24 correlated with invasiveness and differentiation in colorectal adenocarcinoma World J Gastroenterol 2009 15 2258 2264 10.3748/wjg.15.2258 19437567 PMC2682242

[B35] Ghani FI Yamazaki H Iwata S Okamoto T Aoe K Okabe K Mimura Y Fujimoto N Kishimoto T Yamada T Xu CW Morimoto C Identification of cancer stem cell markers in human malignant mesothelioma cells Biochem Biophy res Comm 2011 404 735 742 10.1016/j.bbrc.2010.12.054 21163253

[B36] Pries R Wittkope N Trenkle T Nitsch SM Wollenberg B Potential stem cell marker CD44 is constitutively expressed in permanent cell lines of head and neck cancer In vivo 2008 22 89 92 18396788

[B37] Mack B Gires O CD44s and CD44v6 expression in head and neck epithelia PlosOne 2008 3 10 10.1371/journal.pone.0003360 PMC2566597 18852874

[B38] Liebertz DJ Lechner MG Masood R Sinha UK Han J Puri RK Correa AJ Epstein AL Establishment and characterization of a novel head and neck squamous cell carcinoma cell line USC-HN1 Head Neck Oncol 2010 2 5 10.1186/1758-3284-2-5 20175927 PMC2841166

[B39] Chaffer CL Bruechmann I Scheel C Kaestli AJ Wiggins PA Rodrigues LO Brooks M Reinhardt F Su Y Polyak K Arendt LM Kuperwasser C Bierie B Weinberg RA Normal and neoplastic nonstem cells can spontaneously convert to a stem-like state PNAS 2011 108 7950 7955 10.1073/pnas.1102454108 21498687 PMC3093533

