# Supplementary material for: Identification and characterization of cancer stem cells in human head and neck squamous cell carcinoma
Source: BMC Cancer. 2014 Mar 11;14:173. doi: 10.1186/1471-2407-14-173 (PMC4008349; doi:10.1186/1471-2407-14-173)
Supplement: Additional file 1: Table S1 — Selected gene primers for qRT-PCR. [file 1471-2407-14-173-S1.pdf]

Table 1S. Selected gene primers for qRT-PCR

Target	Forward Primer	Reverse Prime
ALDH1	TCACAGGATCAACAGAG	GTAGAATACCCATGGTGTGC
BMI1	AGGTGACACTATAGAATACAGAAATGCATCGAACAACG	GTACGACTCACTATAGGGAGCTGGTCTCCAGGTAACGAA
Nanog	AGGTGACACTATAGAATATCAGCACCTACCTACCCCAG	GTACGACTCACTATAGGGAGAATTTGGCTGGAACTGCAT
CD133	AGGTGACACTATAGAATAGTGAAAACTAGCCTGCGGTC	GTACGACTCACTATAGGGAGGCCATCCAAATCTGTCCTA
Oct3/4	CTTGCTGCAGAAGTGGGTGGAGGAA	CTGCAGTGTGGGTTTCGGGCA
Sox2	ATGTACAACATGATGGAGACG	GCGCTTGCTGATCTCCGAGT
